# Adult Body Weight Is Programmed by a Redox-Regulated and Energy-Dependent Process during the Pronuclear Stage in Mouse

**DOI:** 10.1371/journal.pone.0029388

**Published:** 2011-12-28

**Authors:** Bernadette Banrezes, Thierry Sainte-Beuve, Eugénie Canon, Richard M. Schultz, José Cancela, Jean-Pierre Ozil

**Affiliations:** 1 INRA, UMR1198 Biologie du développement et Reproduction, Jouy-en-Josas, France; 2 ENVA, Maisons Alfort, France; 3 Department of Biology, University of Pennsylvania, Philadelphia, Pennsylvania, United States of America; 4 Centre de Neurosciences Paris-Sud (CNPS) CNRS UMR 8195 Université Paris Sud, Orsay, France; Istituto Dermopatico dell'Immacolata, Italy

## Abstract

In mammals fertilization triggers a series of Ca^2+^ oscillations that not only are essential for events of egg activation but also stimulate oxidative phosphorylation. Little is known, however, about the relationship between quantitative changes in egg metabolism and specific long-term effects in offspring. This study assessed whether post-natal growth is modulated by early transient changes in NAD(P)H and FAD^2+^ in zygotes. We report that experimentally manipulating the redox potential of fertilized eggs during the pronuclear (PN) stage affects post-natal body weight. Exogenous pyruvate induces NAD(P)H oxidation and stimulates mitochondrial activity with resulting offspring that are persistently and significantly smaller than controls. Exogenous lactate stimulates NAD^+^ reduction and impairs mitochondrial activity, and produces offspring that are smaller than controls at weaning but catch up after weaning. Cytosolic alkalization increases NAD(P)^+^ reduction and offspring of normal birth-weight become significantly and persistently larger than controls. These results constitute the first report that post-natal growth rate is ultimately linked to modulation of NAD(P)H and FAD^2+^ concentration as early as the PN stage.

## Introduction

Fertilized mammalian eggs are sensitive to perturbations in metabolism that can become manifest following birth [1]–[3], but the linkage between early quantitative changes in metabolism and specific long-term effects is poorly understood. Fertilization triggers a series of repetitive Ca^2+^ oscillations that last for several hours and cease around the time of pronuclear (PN) formation [4], [5]. The regime of Ca^2+^ signals drives a temporal set of events that constitute egg activation, including cortical granule exocytosis, cell cycle resumption, and dynamics of PN formation [6]–[8]. Manipulating the regime of Ca^2+^ oscillations can result in long-term effects, *e.g.*, altering the transcriptome in blastocysts and size of offspring [9], [10]. Such findings provide further support that the Developmental Origins of Health and Disease hypothesis (DOHaD) [11] can be extrapolated back to the initial period following fertilization when the egg metabolism is set off [12].

In various mammalian species different types of in-vitro stress can compromise the survival rate [13] and lead to either a reduction [14] or increase in fetal growth [3]. However, if stress affects embryo viability, it remains unknown whether metabolic perturbations during fertilization could impact post-natal growth of offspring. This study was designed to ascertain whether post-natal growth rate could be experimentally manipulated by selectively varying egg metabolism. Such a linkage between early egg metabolism and post-natal phenotype would provide an important experimental model for understanding DOHaD. Here we focus on two metabolic parameters *i.e.*, the cellular redox state (NAD(P)H) and mitochondrial activity (FAD^2+^), which are linked to the regime of Ca^2+^ oscillations triggered by fertilization [12].

The intracellular redox state describes a complex interaction of the relative concentration of reduced and oxidized forms of a variety of molecules including the nicotinamide adenine nucleotides (NAD(P)^+^/NAD(P)H) and flavins (FAD^2+^/FADH_2_). NAD(P)H electron carriers can impact gene expression through the action of several metabolic sensors, e.g., the Carboxyl-terminal Binding Proteins (CtBPs) [15] or Sirtuins (SIRT) [16], [17], which can effect chromatin structure [18]. Variations in the redox potential may play an important role in regulating developmental processes in plants and metazoans [19]–[21] but the relationship between redox state in mammalian eggs and development remains unknown.

Following fertilization, the dynamics of Ca^2+^ oscillations are linked to energy metabolism by stimulating mitochondrial ATP production via oxidative phosphorylation, and indeed, egg activation is accompanied by an increase in intracellular ATP concentration [22]. Ca^2+^ released into the cytoplasm enters the mitochondrial matrix and activates the TCA cycle. During oxidative phosphorylation, a series of redox reactions transfers electrons initially from nutrients to NAD^+^/FAD^2+^, with the reduced forms in turn donating electrons to electron transport chain carriers located in the inner mitochondrial membrane, the final electron acceptor being oxygen [19]. The energy harvested during these redox reactions is stored in an electrical and proton gradient across the inner mitochondrial membrane that drives ATP formation [23]. Mitochondrial ATP production in turn is essential to sustain the Ca^2+^ oscillations that trigger events of egg activation [24].

Fertilized eggs can use exogenous pyruvate or lactate as an energy source; pyruvate serves as a cytoplasmic oxidant and mitochondrial reducing agent whereas lactate serves as a cytoplasmic reducing agent. NADH-mediated reduction of pyruvate consumes a proton, whereas NAD^+^-mediated oxidation of lactate generates a proton, and therefore is linked to intracellular pH (pH_i_). Thus, modest fluctuations in the exogenous pyruvate/lactate ratio or pH can alter the energetic status and cytosolic redox state simultaneously, each of which can alter viability and fetal growth [25].

To establish a connection between redox state in zygotes and the consequences on developmental outcomes following birth, it is essential that the perturbation has minimal effect on development to term. We exploited our ability to drive precisely and specifically the “redox” profile and mitochondrial activity by changing the lactate/pyruvate ratio or pH_i_ of the culture medium [23], [24], [26] during the one cell stage with virtually no impact on development to term (see Results for further description of experimental rationale). We report here for the first time that post-natal growth rates can be up or down regulated by experimentally manipulating NAD(P)H and FAD^2+^ signaling as early as the PN stage following fertilization.

## Results

### Experimental Rationale and Design

By exposing fertilized eggs to exogenous pyruvate, lactate or pH we experimentally manipulated the redox state of the cell, but only within a limited time period during which the fertilized egg partially uses its own stores of energy (*e.g.*, amino acids, carbohydrates, fatty acids). To assess the potential role of pyruvate and lactate we chose a simple M16 medium over a more complex medium, *e.g.*, KSOM/AA to maximize the impact of these treatments on the redox state. We then observed how a transient alteration of the redox state governed by a single exogenous carbohydrate, impacted growth and body weight of adult animals that developed from the manipulated fertilized egg, all other experimental parameters being equal.

We used NAD(P)H and FAD^2+^ auto-fluorescence as a proxy for metabolic changes [19], [27], [28], and designed four culture media with different carbohydrate compositions and pH to vary preferentially the mitochondrial activity (FAD^2+^ signal) and the redox state (NAD(P)H signal). A micro-fluidic approach was employed to record precisely the kinetics of the auto-fluorescence signal during the PN stage. The treated embryos were transferred into foster mothers at the 2-cell stage and the growth profile of the offspring was determined until the 22^nd^ week of age as a phenotypical trait that reveals the long-term influence of egg metabolism.

### Energy starvation causes NAD(P)H oxidation and low mitochondrial activity, and offspring, which are small before weaning, tend to catch up thereafter

In mouse eggs, protein degradation accelerates shortly after fertilization and can provide a degree of energy autonomy for a few hours [29], [30]. Therefore, we first evaluated how fertilized eggs survived and developed to term following a 10 or 15 h period of starvation during the PN stage. We then correlated the egg NAD(P)H and FAD^2+^ profiles with the growth profiles of the offspring.

**Figure 1:**
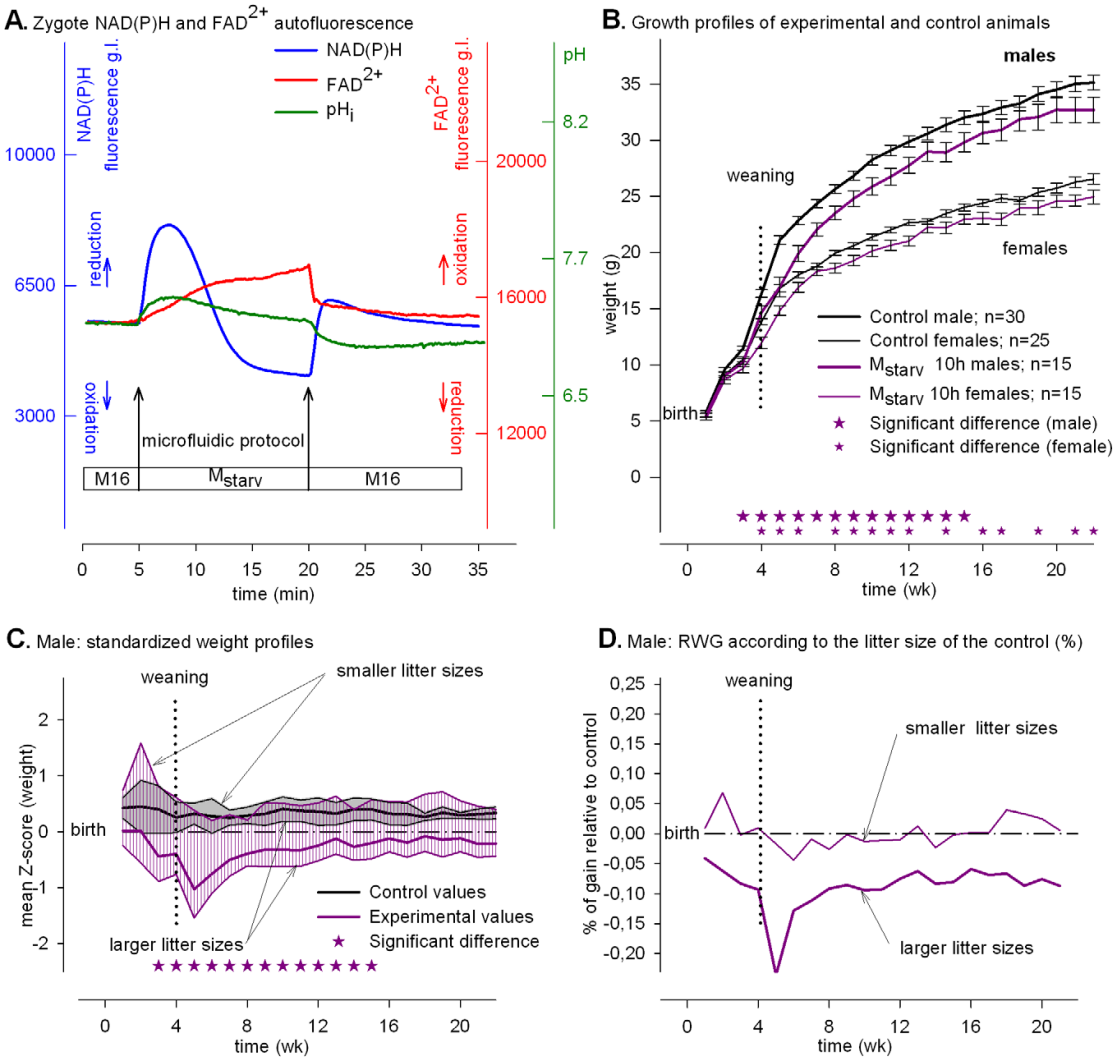
Energy starvation and developmental responses. Metabolic profiles and post-natal phenotypes induced by “starvation.” (**A**) Time course of intracellular NAD(P)H (blue), FAD^2+^ (red) signals (grey levels, g.l., 14 bits) and pH_i_ fluorescence (green) of eggs subjected to M_starv_. The initial NAD(P)H biphasic curve is due to the high lactate/pyruvate ratio in M16 that delays intracellular depletion of carbohydrates. (**B**) Growth profiles of control (black lines) and experimental animals (dark pink lines). The data are expressed as mean ± SEM. (**C**) Standardized plots of the total Z values (n = 210). The upper limits of the shaded zones (grey for controls and dark pink for the experimental values) are the normalized weight profiles of males issued from the larger litter sizes (8, 7 and 6) and the lower limits, from the smaller litter sizes (5, 4, 3 and 2). (**D**) Relative weight gains (RWG) of experimental animals according to the larger or smaller litter size group. In this figure and in figures 2–4, the stars in panels B and C denote significant differences between average experimental and control values (with a P-value at least <0.04) when compared to controls.

Depleting exogenous carbohydrates induced a biphasic change of the NAD(P)H signal (**Fig. 1A**, blue line). This biphasic signal revealed that a virtually instant replacement of M16 by M_starv_ induced an initial increase of NAD(P)H fluorescence followed by a rapid decline when intracellular carbohydrates were totally washed out. The increase in the FAD^2+^ signal, which is of mitochondrial origin [12] (**Fig. 1A**, red line) and simultaneously monitored, suggested that mitochondrial activity, *i.e.*, ATP production, decreased. During starvation for 10 or 15 h, both NAD(P)H and FADH_2_ became more oxidized (**Fig. S1**). The transient change in pH_i_ was due to sodium/proton equilibration due to replacing sodium lactate/pyruvate and glucose with NaCl to maintain osmolarity (**Fig. 1A**, green line).

Despite an elevated rate of NAD(P)H and FADH_2_ oxidation, the incidence of cleavage to the 2-cell stage after 10 h of starvation was 65.2%, and 57.1% of embryos developed to term after embryo transfer (**Table 1**). In contrast, after 15 h of starvation, only 10.2% zygotes cleaved to the 2-cell stage, and of those only 30.7% developed to term (**Table 1**). Hence, the window of energy autonomy progressively declined between 10 and 15 h of starvation.

**Table 1:** Developmental potential of experimental and control eggs.

Treatment	# Egg	# 2-C (%)	# Recipient	# 2-C transferred number^a^	# Pup	Survival (%)	Litter size±SEM
**Control**	351	351 (100)	11	88	56 ^(1^ † ^w2)^	63.6	5.1±0.5
**M_starv_ 10 h**	164	107 (65.2)*	7	56	32 ^(2^ † ^w1; w8)^	57.1	4.6±0.9
**M_starv_ 15 h**	234	24 (10.2)*	2	13	4	30.7**	2±0.0
**M_pyr_**	157	157 (100)	7	56	46	82.1	6.6±0.7
**M_lac_**	179	177 (98.9)	8	64	47 ^(2^ † ^w1; w9)^	73.4	5.9±0.4
**M_pH_**	91	90 (98.9)	6	48	32 ^(2^ † ^w1; w3)^	66.7	5.3±0.6
**Total**	1176	907	41	325	217	66.7	5.5±0.3

# = number.

* and ** denotes significant differences.

*The rate of two-cell of the M_starv_ 10 and 15 hours are significantly lower than the other groups, Chi-square test (p<0.001).

**The rate of survival of the M_starv_ 15 hours are significantly lower than the pyruvate group, Chi-square test (p = 0.017).

† Dead newborns at week n: w(n). 8 eggs were transferred for every recipient except for the M_starv_ 15 h group.

Both male and female offspring derived from zygotes starved for 10 h were significantly smaller (dark pink lines) than controls (black lines) by the time of weaning (**Fig. 1B**). The impact of litter size was evaluated by standardizing the data using the Z-score method (normalized data, see Materials and Methods). For males, the normalized growth profiles (Z-score) of experimental and control animals issued from the smaller litter sizes were similar, *i.e.*, the highest limits of the shaded dark pink (experimental) and grey (control) zones were similar (**Fig. 1C**). In contrast, the Z-score profiles of experimental animals derived from larger litter sizes were below that of the controls, *i.e.*, the lowest limit of the shaded dark pink zone (experimental) was beneath the lowest limit of the grey zone (control) (**Fig. 1C**). For females, the Z-score growth profiles although of smaller amplitude, display similar tendency (**Fig. S2A**). The effects induced by larger litter sizes during pregnancy amplified this negative effect on weight gain relative to control for both males and females (relative weight gain: RWG, **Figs. 1D** **and S2B**).

### Exogenous pyruvate induces NAD(P)H oxidation and stimulates mitochondrial activity and offspring are persistently smaller after weaning

To discriminate further whether high oxidation of cytosolic NAD(P)H or low mitochondrial activity (high FADH_2_ oxidation) was responsible for the low post-natal growth, we cultured fertilized eggs for 15 h (the limit of energy autonomy) during which pyruvate was the sole energy source; pyruvate stimulates mitochondrial activity (*i.e.*, maintaining a balanced FADH_2_/FAD^2+^ ratio) but oxidizes the cytosolic NAD(P)H [24] as does starvation.

In mouse eggs, pyruvate serves as the main metabolite oxidized inside mitochondria for energy production, but is also reduced in the cytosol by the lactate dehydrogenase (LDH) with concomitant oxidation of NADH [19], [22], [31]. Pyruvate as the sole exogenous source of carbohydrate, however, may compromise pre-implantation development [24]. Accordingly, we evaluated survival to term and post-natal impact when fertilized eggs were exposed for only 15 h to the cytosolic NADH oxidant action of pyruvate.

**Figure 2:**
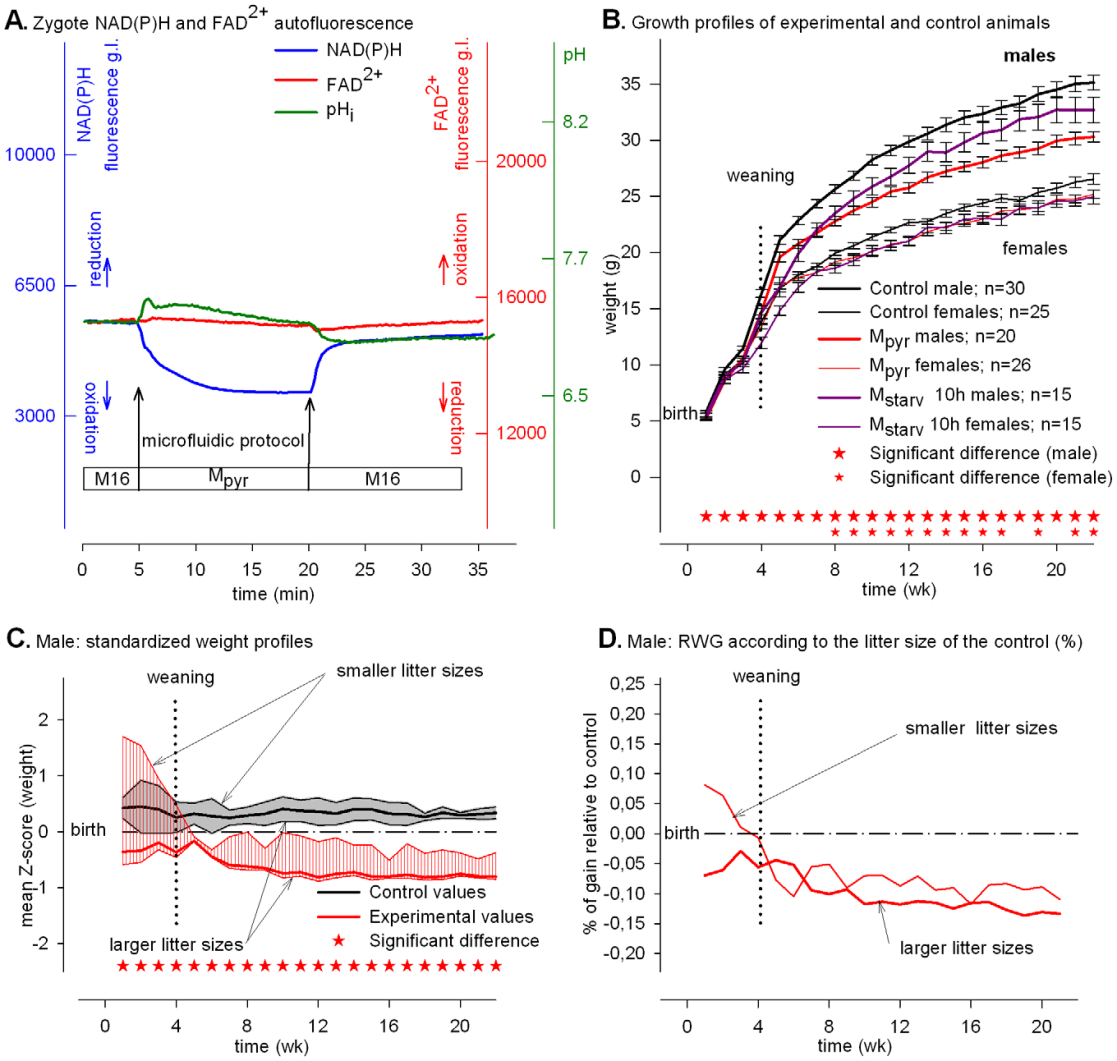
Exogenous pyruvate and developmental responses. Metabolic profiles and post-natal phenotypes induced by “pyruvate.” (**A**) Time course of intracellular NAD(P)H (blue), FAD^2+^ (red) signal (grey levels, g.l., 14 bits) and pH_i_ fluorescence (green) of eggs subjected to M_pyr_. (**B**) Growth profiles of control (black lines) and experimental (red lines) animals. The growth profiles of M_starv_ animals from **Fig. 1B** are plotted as dark pink lines for comparison. The data are expressed as mean ± SEM. (**C**) Standardized plots of total Z values (n = 210). The upper limits of the shaded zones (grey for the controls and red for the experimental values) are the normalized weight profiles of males issued from the larger litter sizes (8, 7 and 6) and the lower limits, from the smaller litter sizes (5, 4, 3 and 2). (**D**) Relative weight gains (RWG) of experimental animals according to the larger or smaller litter size group.

Pyruvate alone caused an immediate decrease of the NAD(P)H signal (**Fig. 2A**, blue line), whereas the FAD^2+^ signal (**Fig. 2A**, red line) remained unchanged as was pH_i_ (**Fig. 2A**, green line). After 15 h in M_pyr_, the level of NAD(P)H oxidation remained low and the FAD^2+^ signal remained at the resting level (**Fig. S3**). Hence, pyruvate supported a higher rate of cytosolic NAD(P)H oxidation and mitochondrial activity was not impaired by prolonged culture (15 h).

All treated fertilized eggs cleaved to the two-cell stage and following embryo transfer developed to term at a high incidence (82.1%, **Table 1**). Nevertheless, the post-natal growth profiles of animals of both genders (red lines) were significantly smaller than controls (back lines) but with the males also significantly smaller than those issued from starvation (dark pink lines) (**Fig. 2B**). The animals were healthy but remained small. This effect did not appear related to litter size because whatever the litter size, the normalized growth profiles (Z-score) of the higher and lower limits of the shaded red zone (experimental) remained below the upper and lower limits of the grey zone (control) (**Fig. 2C** for males and **Fig. S4A** for females). Hence, contrary to “Starvation,” whatever the litter sizes, the RWG following “Pyruvate” remained persistently low for both genders (**Fig. 2D** **and S4B**).

### Exogenous lactate stimulates NAD^+^ reduction and impairs mitochondrial activity, and offspring that are small at weaning catch up afterward

To address whether the aforementioned phenotype could be changed by treatments that reverse the redox potential of the NAD^+^ during the same period of time, we next cultured fertilized eggs in medium in which the sole energy source was lactate. In contrast to pyruvate, lactate is poorly metabolized by the mitochondria but is a strong cytosolic reductant via LDH activity [24]. Hence, we evaluated how offspring growth would be affected by a high rate of NAD^+^ reduction and lactate metabolism within the 15 h time frame.

**Figure 3:**
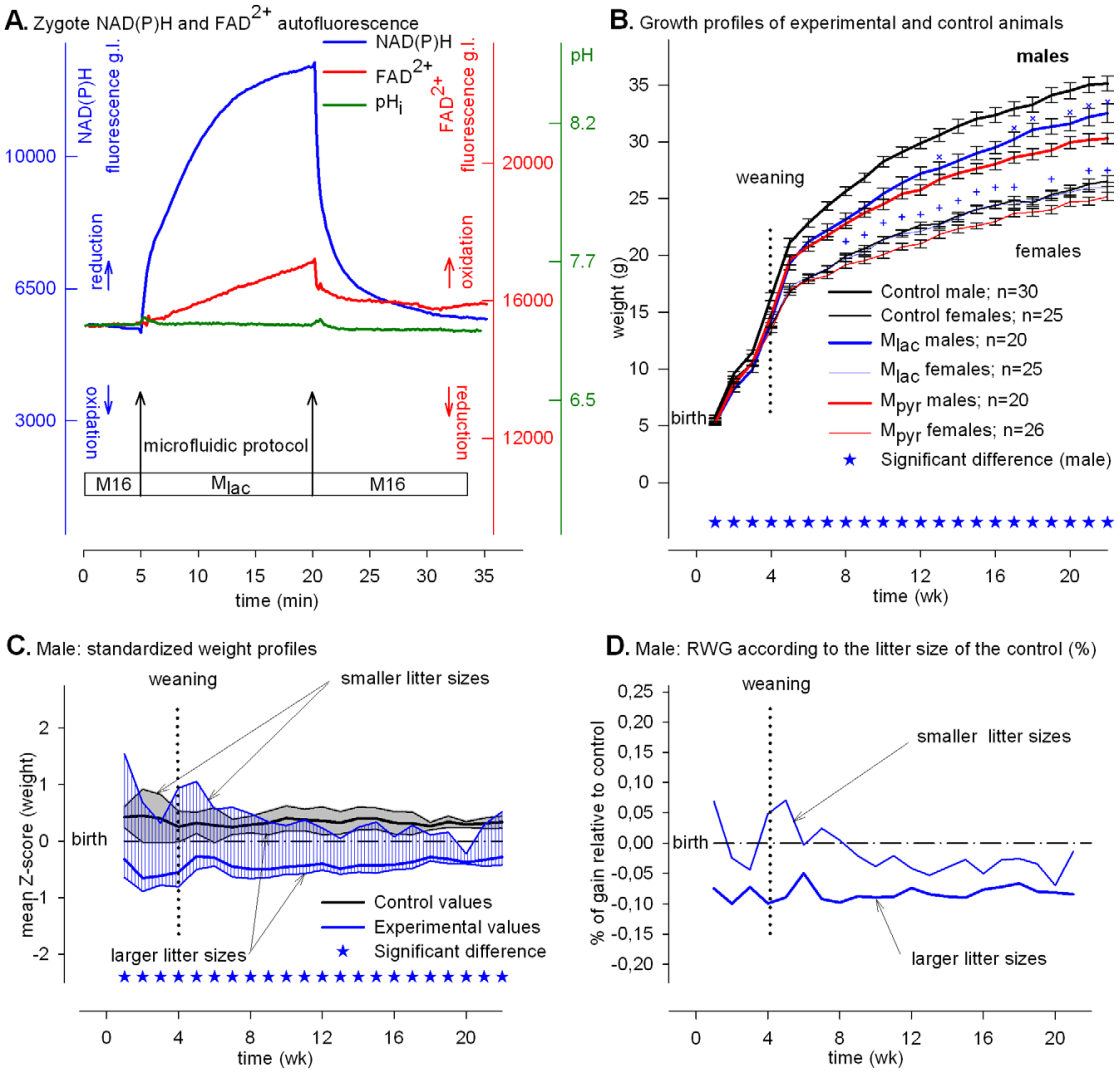
Exogenous lactate and developmental responses. Metabolic profiles and post-natal phenotypes induced by “Lactate.” (**A**) Time course of intracellular NAD(P)H (blue), FAD^2+^ (red) signals (grey levels, g.l., 14 bits) and pH_i_ fluorescence (green) of eggs subjected to M_lac_. (**B**) Growth profiles of control and experimental animals. The growth profiles of M_pyr_ animals from **Fig. 2B** are plotted as red lines for comparison. The blue crosses and plus symbols show when the M_lac_ profiles are significantly higher than the M_pyr_ profiles for males and females, respectively. The data are expressed as mean ± SEM. (**C**) Standardized plots of total Z values (n = 210). The upper limits of the shaded zones (grey for the controls and blue for the experimental values) are the normalized weigh profiles of males issued from the larger litter sizes (8, 7 and 6) and the lower limits from the smaller litter sizes (5, 4, 3 and 2). (**D**) Relative weight gains (RWG) of experimental animals according to the larger or smaller litter size group.

Replacing M16 with M_lac_ induced an abrupt and simultaneous steep increase of NAD(P)H and FAD^2+^ signals indicating that mitochondrial activity was virtually instantly down-regulated in the presence of lactate (**Fig. 3A** **,** blue and red lines); note that pH_i_ was not altered by the treatment (**Fig. 3A**, green line). These two signals remained elevated at a constant level for 15 h (**Fig. S5**). Although the FAD^2+^ signal indicated that mitochondrial activity was impaired during the treatment, almost all of the fertilized eggs cleaved to the 2-cell stage (**Table 1**). In addition, the high incidence of survival to term (73.4%, **Table 1**) suggests that lactate provided an additional source of energy that may or may not be related to some level of lactate-derived pyruvate production [24]. Nevertheless, the growth of males (but not females) was significantly lower (blues lines) than that of the controls (black lines) but significantly higher than that following the “Pyruvate” treatment (red lines) (**Fig. 3B**, blue×symbol). The normalized growth profiles (Z-score) of experimental and control animals derived from the group of smaller litter sizes (5, 4, 3 and 2) were very similar, *i.e.*, the upper limits of the shaded blue (experimental) and grey (control) zones overlap before weaning and at adult age (**Fig. 3C** for males and **Fig. S6A** for females). In contrast, the Z-score profiles of experimental male animals derived from the group of larger litter sizes (8, 7 and 6) were below those of the controls, *i.e.*, the lowest limit of the stippled blue zone (experimental) was beneath the lowest limit of the grey zone (control) (**Fig. 3C**). No litter size effect was observed for the females (**Figs. S6A** and **B**). For males, this effect appeared related to litter size because the RWG of males derived from smaller litter sizes was higher than that for males derived from larger litter sizes (**Fig. 3D**). Unlike the “Pyruvate” treatment, larger litter sizes during pregnancy have a tendency to amplify a negative effect of “Lactate” at least for the males. The growth profiles for females were similar to those of the controls but significantly higher than “Pyruvate” (red line) (**Fig. 3B**, blue+symbol). Hence, elevated NAD^+^ reduction caused by lactate reversed the tendency observed with high oxidation by pyruvate for both genders but without reaching that of the control.

### Cytosolic alkalization increases NAD(P)^+^ reduction and offspring that are normal at birth become persistently large after weaning

To circumvent the impact on mitochondrial activity when lactate was the sole energy source, we developed a procedure that consisted of first culturing fertilized eggs in standard M16 culture media to maintain the normal supply of exogenous carbohydrates, and then transiently increased the external pH to down-regulate the oxidative activity of the mitochondria by dissipating the proton gradient outside the mitochondrion matrix that is known to correlate with NAD(P)H increase in sea urchin eggs [32], [33].

**Figure 4:**
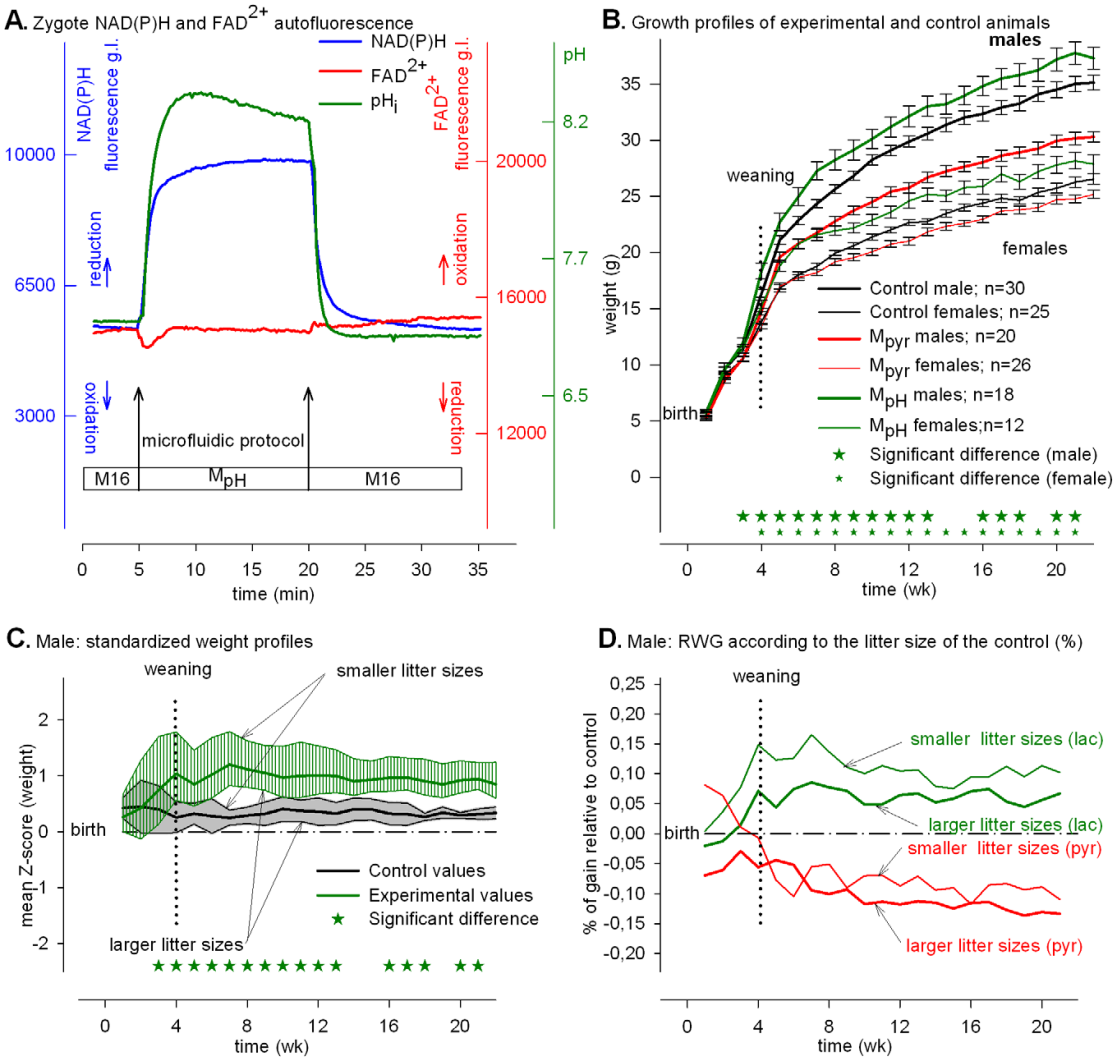
Alkalization and developmental responses. Metabolic profiles and post-natal phenotypes induced by “alkalization.” (**A**) Time course of intracellular NAD(P)H (blue), FAD^2+^ (red) signals (grey levels, g.l., 14 bits) and pH_i_ fluorescence (green) of eggs subjected to M_pH_. (**B**) Growth profiles of control and experimental animals. The growth profiles of M_pyr_ animals from **Fig. 2B** are plotted as red lines for comparison. The data are expressed as mean ± SEM. (**C**) Standardized plots of total Z values (n = 210). The upper limits of the shaded zones (grey for the controls and green for the experimental values) are the normalized weight profiles of males issued from the larger litter sizes (8, 7 and 6) and the lower limits from the smaller litter sizes (5, 4, 3 and 2). (**D**) Relative weight gains (RWG) of M_pH_ experimental animals according to the larger or smaller litter size group (green lines). The RWG of M_pyr_ animals from **Fig. 2B** are plotted as red lines for comparison.

Fertilized eggs cultured in M16 culture media loaded with NaOH, which resulted in a pH value of 8.2, displayed steep increase in pH_i_ (**Fig. 4A**, green line) that reached a plateau within a few minutes. The NAD(P)H signal (**Fig. 4A**, blue line) increased virtually instantaneously whereas the FAD^2+^ signal (**Fig. 4A**, red line) remained essentially unchanged, showing that the balance between reduction and oxidation of FADH_2_/FAD^2+^ inside the mitochondrion matrix remained balanced at a high pH_i_.

The pH_i_-dependent rise in NAD(P)H was similar to that observed in sea urchin eggs following fertilization [32], [33]. The kinetics of the proton/hydroxide equilibrium between the outside and inside of the fertilized egg was very fast and highly reversible (**Fig. 4A**), and might involve translocation of protons across the plasma membrane [34] that bypasses the fertilized egg's normal regulative processes [35]–[37]. Although we cannot dissociate the impact of pH_i_ from the changes in NAD(P)H, the NAD(P)H signal did not remain high, but decreased within 3–5 h to the resting level (**Fig. S7**).

Under these conditions, all of the fertilized eggs cleaved to the 2-cell stage and 66.7% of them developed to term (**Table 1**). These results demonstrate that a transient rise in pH_i_ and NAD(P)H during the PN stage did not compromise development to term but the growth of both genders (green lines) was significantly accelerated immediately after birth in comparison to the controls or to “Pyruvate” (red lines) (**Figs. 4B and C**). This effect did not appear related to litter size because after weaning, whatever the litter size, the normalized growth profiles (Z-score) of the upper and lower limits of the shaded green zone (experimental) remained above the highest limit of the grey zone (control) (**Fig. 4C** for males and **Fig. S8A** for females). Like the “Pyruvate” treatment, but unlike the “Starvation” and “Lactate” treatments, the RWG was not affected by litter size for both genders (**Figs. 4D** **and S8B**).

## Discussion

**Figure 5:**
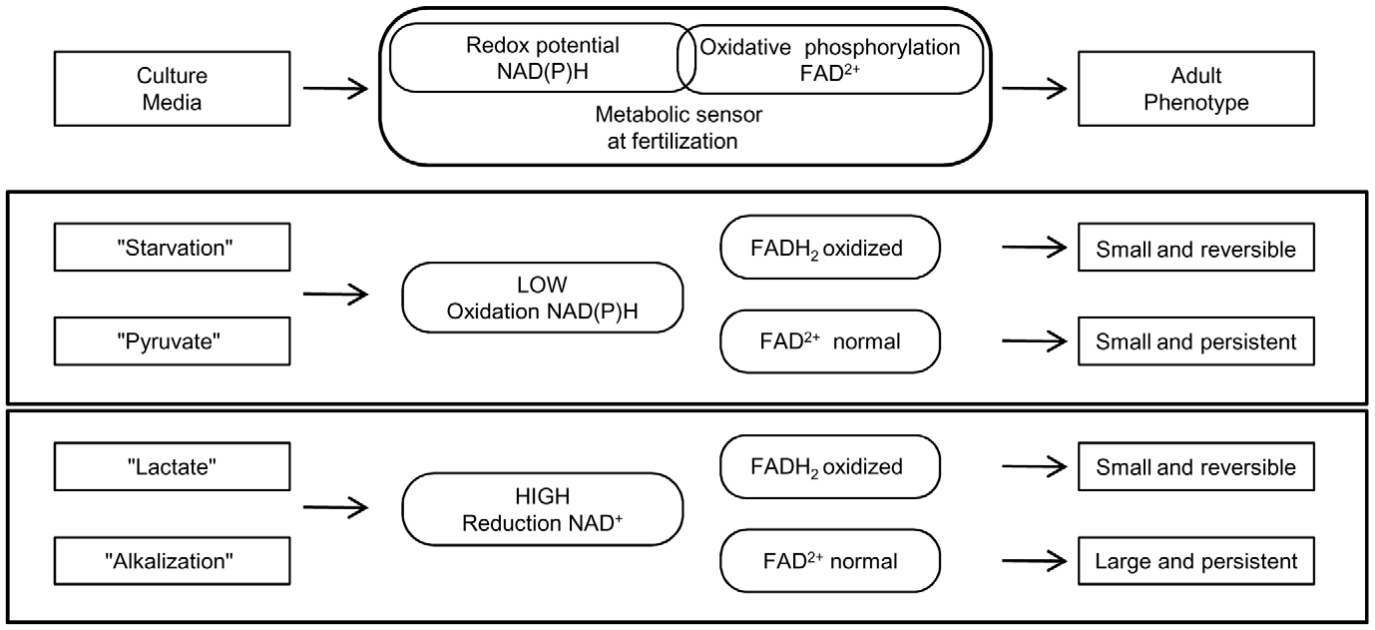
Post-natal outcomes resulting from zygotic metabolic sensor activities. The diagram represents the linkages between the composition of the culture media, the NAD(P)H and FAD^2+^ concentrations and the adult phenotype, that are mediated by a zygotic metabolic sensor.

We report here that experimentally manipulating the redox potential in fertilized eggs during the PN stage does not compromise viability but has long-term consequences that become manifest on post-natal growth rates (summarized in **Fig. 5**). All else being equal—embryo production, pregnancy length restricted to 19 days, and animal breeding—when egg mitochondrial activity is compromised by the absence of exogenous pyruvate, *i.e.*, with “Starvation” and “Lactate” treatments, eggs in both cases develop to term but the offspring display a smaller post-natal growth rate than “Control”, mostly for the males (**Figs 1B** and **3B**). Thus, eggs can survive a transient degree of energetic depression during the PN stage (**Table 1**) but at the cost of post-natal growth. Moreover, the high or low NAD(P)H redox level imposed by “Starvation” or “Lactate” does not appear to have long-term consequences because the growth of animals tends to catch up whatever the redox potential (**Figs 1** and **3**). In contrast, when mitochondrial activity is supported by pyruvate in the culture media, post-natal growth profile varies as a function of high or low redox potential. When cytosolic NAD(P)H oxidation is set high (low reducing power) by “Pyruvate” treatment the adults (males and females) are smaller, whereas when cytosolic NAD(P)H oxidation is set low (high reducing power) by transient cellular alkalization the growth rate accelerates and results in larger adult animals (see **Figs. 4B and 4D** where the growth profiles of “Pyruvate”, “Control” and “Alkalization” are superimposed). Because the experimental design provides a high survival rate to term (**Table 1**) it is possible to observe a strong linkage between transient changes in egg metabolism (redox and mitochondrial activity) and post-natal development. Our finding that male and female offspring display different levels of sensitivity to the treatments presumably reflects differences in their chromosomal complement and physiology [38]. The present results underscore previous findings that the gender difference is an intriguing feature of developmental programming [39].

We also find that when egg metabolism is compromised by “Starvation” and “Lactate” treatments, the growth profile in both cases depends on litter size. Mice from smaller litter sizes have a normal RWG whereas mice from larger litter sizes have a smaller RWG (Figs. **1D** and **3D**). Thus, when maternal resources per fetus are sufficient during fetal life (*i.e.*, small litter size), eggs experiencing “Starvation” or “Lactate” treatments recuperate and a normal growth rate occurs during their fetal life. Thus, a transient metabolic impact during the PN stage is reversible and sensitive to a litter size effect during fetal life whatever the redox potential during the treatment. In contrast, when egg mitochondrial activity is not impaired by the absence of exogenous pyruvate, the growth profile is insensitive to litter size whatever the redox potential. With “Pyruvate” treatment, mice from either smaller or larger litter sizes have a smaller RWG (**Figs. 2D** **and S4B**), whereas with the “Alkalization” treatment, mice from lower or higher litter sizes both have a higher RWG (**Figs. 4D** **and S8B**). Thus, when egg mitochondrial activity is not impaired during fertilization, the impact on the RWG is not prone to changes in relation to the maternal resources during pregnancy, *i.e.*, litter size. In these experimental situations (“Pyruvate” and “Alkalization” treatments), the long-term impact appears set at the PN stage and remains insensitive to litter size during fetal life, with a permanent impact of a high or low redox potential. The high or low redox states have different post-natal outcomes at the adult age depending upon mitochondrial activity that appears to be a key actor in setting either long or short-lasting responses. One simple hypothesis for this observation is that a highly sensitive metabolic sensor, which remains to be identified, translates the mitochondrial activity and modulates the long term responses as outlined in **Fig. 5**.

Starvation and lactate treatments lead to low mitochondrial activity of the egg that is impaired because the FAD^2+^ signal immediately increases (FADH_2_ becomes more oxidized) and stays high during the treatments (**Figs. 1A** and **3A**). That a high or low NADH redox potential results in similar post-natal growth (small offspring whose growth catches up later in life) indicates that low mitochondrial activity during the treatment overrides the potential long-term effect of the NAD(P)H redox potential. In contrast, pyruvate treatment does not compromise the mitochondrial activity but results in permanently small animals, whereas “Starvation”, which also causes NADH oxidation, results in small animals that catch up after weaning.

It is likely that the “Alkalization” treatment at least partially impairs mitochondrial activity energy production by dissipating the mitochondrial proton gradient (**Fig. 4A**) [23]. Nevertheless, the energy status of the cell is likely to be far less compromised than for the starvation treatment since the survival rate at the 2-cell stage is identical to the controls but higher than those issued from starvation (Table 1). This is probably due to the presence of exogenous pyruvate during alkalization that helps the egg to cover its energy demand during the treatment and the short duration of the mitochondrial perturbation due to the rapid restoration of the resting pH_i_ level by the buffering action of bicarbonate in the presence of CO_2_ (see **Fig. S7**). In this situation, the transient increase in NAD(P)H signal (high reducing power) is capable of accelerating post-natal growth. Moreover, the intensity of the post-natal growth suggests that the treatment requires only a short period of a few hours to provoke a long lasting impact.

The results described here also provide further evidence that post-natal weight variation is sensitive to changes in the Ca^2+^ regime at fertilization [40], which increase mitochondrial activity and thus augments reducing equivalents of NADH and FADH_2_ [19]. Our findings support a previous hypothesis suggesting that an electrochemical sensor(s) is capable of influencing developmental processes according to small changes in egg metabolism at the time of fertilization [19], [41], [42]. At this juncture, however, it is difficult to ascribe a molecular mechanism that underpins the observed changes in postnatal growth rate. An obvious candidate is failure to recapitulate faithfully the dramatic reprogramming of gene expression during the 2-cell stage; such reprogramming is essential for further development [43]. We have conducted microarray experiments on 2-cell embryos derived from controls and manipulated 1-cell embryos. An unsupervised cluster analysis did not reveal any difference between the control and experimental groups. Moreover, of the few mis-expressed transcripts, the change in relative abundance of affected transcripts was typically within 20% of the control. Finding that reprogramming occurs with relatively high fidelity in the treated embryos is not surprising because we find development to term is unaffected, whereas substantially perturbing the fidelity of reprogramming leads to either developmental arrest at the 2-cell stage or a marked delay in preimplantation development [44].

By manipulating the metabolism of a fertilized egg without perturbing survival to term we have unveiled a surprising linkage between a very brief perturbation in metabolism of a 1-cell embryo and changes in adult phenotype. Exploiting this property has obvious practical applications in animal reproductive biotechnology. The results also have clinical implications regarding use of assisted reproductive technologies (ART) to treat human infertility. Embryo culture commencing at the 1-cell stage is an intrinsic part of ART. Previous studies using a mouse model predicted several years in advance the observed increased risk for ART-conceived children for loss-of-imprinting syndromes [*e.g.*], [ 39,40].

## Materials and Methods

### Ethics Statement

Animal experiments were carried out in strict accordance with the recommendations in the guidelines of the Code for Methods and Welfare Considerations in Behavioural Research with Animals (Directive 86/609EC). All efforts were made to minimize suffering. Experiments were approved by the Committee on the Ethics of Animal Experiments of the author's institution, INRA (permit number 11 024).

### Embryo production and collection

Female mice (C57BL/6×CBA) were superovulated by i.p. injection of 8 IU of PMSG (Syncro-Part® CEVA) followed 48 h later by injection of 7.5 IU of hCG (Chorulon® Intervet) and caged overnight with F1 males. Fertilized eggs were recovered 17 h after hCG injection, and then placed in drops under mineral oil and incubated under an atmosphere of 5% CO_2_ in air at 37°C. Only eggs that displayed two PN were subjected to experimental treatments.

### Culture media and embryo treatment

Culture media were formulated from standard M16 that has the highest lactate to pyruvate ratio [45]. Variation of the NAD(P)H and FAD redox levels was obtained by using a single source of carbohydrate in the culture medium at standard concentration, *i.e.*, 23.28 mM lactate or 0.33 mM pyruvate, which are referred to as M_lac_ and M_pyr_, respectively. Starvation experiments were performed with M16 medium devoid of carbohydrates (M_starv_). The effect of extracellular pH was explored by adding 2 mM NaOH into the M16 medium containing the normal composition of carbohydrates (M_pH_). NaCl concentration was increased in M_starv_, M_lac_ and M_pyr_ to maintain osmolarity. All chemicals were purchased from Sigma and VWR. Fertilized eggs were washed thoroughly and placed in 50 µl drops of experimental media (10–30 embryos per drop) under paraffin oil and were cultured for 10 or 15 h in an atmosphere of 5% CO_2_ in air at 37°C. The 2-cell embryos were then washed in M16 and transferred to pseudo-pregnant recipients. Four experiments and one control were designed. Treatment “Starvation”; eggs were cultured in M_starv_ for 10 or 15 h. Treatment “Pyruvate”; eggs were cultured in M_pyr_ for 15 h. Treatment “Lactate”; eggs were cultured in M_lac_ for 15 h. Treatment “Alkalization”; eggs were cultured in M_pH_ for 15 h. Treatment “Control”; eggs were cultured in M16 for 15 h.

### Intracellular fluorescence recording

Two eggs were held individually in a microfluidic chamber by two holding micropipettes in a viewing field of a Nikon TE2000 inverted microscope fitted with a Fluor 40× oil immersion objective (NIKON) as previously described [40]. The optical field was illuminated with a 75-W Xenon arc lamp (OSRAM) and specific excitation wavelengths were selected from a Cairn Optoscan Monochromator (DIPSI). NAD(P)H autofluorescence was measured by excitation at 365 nm±5 nm. The emission was collected through a band-pass filter (445–45 nm) (Semrock Filter). FAD^2+^ autofluorescence was measured by excitation at 450 nm±16 nm. The emission was collected at 520–35 nm. Fluorescence intensity was captured using the EMCCD QuantEM 521SC (512×512 pixel) cooled camera from Photometric (Roper Scientific) with 300 ms exposure time. The whole process was controlled by the MetaFluor software v7.1 (Roper Scientific). All records are the average value of 6 records and are plotted with SigmaPlot 11 software (Ritme Informatique). For measuring variation of pH_i_, eggs were incubated at 37°C for 15 min in M16 with 20 µM SNARF-1-AM (Invitrogen). SNARF-1 fluorescence was detected with an excitation wavelength of 535 nm and two emission wavelengths of 640 nm and 600 nm [28]. The pH_i_ records are independent of the NAD(P)H/FAD^2+^ records.

### Microfluidic device and method

The microfluidic chamber previously described to drive Ca^2+^ signaling [40] makes possible the ability to change the entire chamber volume (10 µl) in less than 1 second at 37°C. All tubing is gas impermeable (Upchurch-Rheodyne). The media were loaded into the central unit (prototype BRACER; Jouy-en-Josas, France) directly from the incubator (5% CO_2_ in air). Eggs were subjected to three sequences of microfluidic flux. The first sequence makes it possible to record the resting signals in M16; the second, the kinetic of changes induced by the experimental media for 15 min; and the third, the kinetic of changes when M16 is back into the chamber.

### Embryo transfer and animal Phenotyping

Female F1 mice (C57BL/6×CBA), 8–13 weeks old, were used as recipients. Experimental and control eggs at the two-cell stage were transferred in groups of 8 to the left oviduct of each recipient female. Starting the first week after delivery, newborns were weighed every week until the 22^nd^ week. The pups were weaned at the 4^th^ week and males and females in each litter were separated and placed in groups of 5 in independent cages. Mice were fed with a standard laboratory diet and tap water ad-libitum. We calculated the relative weight gain (RWG) as follows: for week n, RWGn is the difference between the mean weight of the experimental and mean weight of the control animals divided by the mean weight of the control animals.

### Statistical analysis

The data were analyzed using SigmaStat 3.0 software. The Chi-square test was used to analyze the rate of cleavage to the two-cell stage, the survival rate at term, the gestation length, the mean litter (Table 1). Growth data were converted to Z-scores prior to statistical analysis. We used SigmaPlot V11 to standardize the entire data set (n = 210 living animals) to the same scale (μ = 0, *σ* = 1). Student's *t* test was used for comparing the Z values of the experimental groups versus the control. *P*<0.05 was considered to be statistically significant. To visualize the impact of litter size on growth better, we also separately plotted the Z-values of animals issued from the larger litter size which are above the average value of litter sizes (5.5 see Table 1) that include 8, 7 and 6 litter sizes, and from the lower litter sizes which are below the average value, *i.e.*, 5, 4, 3 and 2 (only 2 females gave birth to 2 offspring in control and lactate experiments. None of the females delivered a single offspring). Significant differences between correlations were analyzed week by week. For all tests, sample numbers and P values are included in the text.

## Supporting Information

Figure S1 **NAD(P)H and FAD^2+^ profiles from eggs subjected to M_starv_ for 10 or 15 h.** The NAD(P)H oxidation (blue line) induced by starvation in early 20 min is thereafter amplified by prolonged duration for 10 or 15 h. The level of FAD^2+^ (red line) is oxidized in early 20 min and remains oxidized at similar levels for 10 or 15 h. The records of the early 20 min are the copy from those plotted on **Fig. 1A**.
(TIF)

Figure S2 **Z profiles and RWG for females issued from eggs subjected to M_starv_.** (**A**) The upper limits of the shaded zones (grey for controls and dark pink for the experimental values) are the normalized weight profiles of females issued from the larger litter sizes (8, 7 and 6) and the lower limits, from the smaller litter sizes (5, 4, 3 and 2). (**B**) Relative weight gains (RWG) of experimental animals according to the larger or smaller litter size group. In this figure and in figures **S4** and **S8**, the stars in panel **A** denote significant differences between average experimental and control values (with a P-value at least <0.04) when compared to controls.
(TIF)

Figure S3 **NAD(P)H and FAD^2+^ profiles from eggs subjected to M_pyr_ for 10 or 15 h.** The level of NAD(P)H oxidation (blue line), induced by M_pyr_ in early 20 min, remains highly oxidized for 10 or 15 h. The level of FAD^2+^ (red line) remains at a constant level for 10 or 15 h. The records of the early 20 min are the copy from those plotted on **Fig. 2A**.
(TIF)

Figure S4 **Z profiles and RWG for females issued from eggs subjected to M_pyr_.** (**A**) The upper limits of the shaded zones (grey for the controls and red for the experimental values) are the normalized weight profiles of females issued from the larger litter sizes (8, 7 and 6) and the lower limits, from the smaller litter sizes (5, 4, 3 and 2). (**B**) Relative weight gains (RWG) of experimental animals according to the larger or smaller litter size group.
(TIF)

Figure S5 **NAD(P)H and FAD^2+^ profiles from eggs subjected to M_lac_ for 10 and 15 h.** The level of NAD(P)H reduction (blue line), induced by M_lac_ in early 20 min, remains highly reduced for 10 or 15 h. The level of FAD^2+^ (red line) is oxidized in the early 20 min and remains oxidized at a constant level for 10 or 15 h. The records of the early 20 min are the copy from those plotted on **Fig. 3A**.
(TIF)

Figure S6 **Z profiles and RWG for females issued from eggs subjected to M_lac_.** (**A**) The upper limits of the shaded zones (grey for the controls and shaded blue for the experimental values) are the normalized weight profiles of females issued from the larger litter sizes (8, 7 and 6) and the lower limits, from the smaller litter sizes (5, 4, 3 and 2). (**B**) Relative weight gains (RWG) of experimental animals according to the larger or smaller litter size group.
(TIF)

Figure S7 **NAD(P)H and FAD^2+^ profiles from eggs subjected to M_pH_ for 3 h.** The records show that the NAD(P)H level induced by M_pH_ by the early 20 min, declines rapidly thereafter and reaches the resting level in a couple of hours. The level of FAD^2+^ remains constant.
(TIF)

Figure S8 **Z profiles and RWG for females issued from eggs subjected to M_pH_.** (**A**) Standardized plots of total Z values (n = 210). The upper limits of the shaded zones (grey for the controls and green for the experimental values) are the normalized weight profiles of females issued from the larger litter sizes (8, 7 and 6) and the lower limits, from the smaller litter sizes (5, 4, 3 and 2). (**B**) Relative weight gains (RWG) of M_pH_ experimental animals according to the larger or smaller litter size group (green lines). The RWG of M_pyr_ females from **Fig. S4** are plotted as red lines for comparison.
(TIF)
