# Supplementary figures and images for: Adult Body Weight Is Programmed by a Redox-Regulated and Energy-Dependent Process during the Pronuclear Stage in Mouse
Source: PLoS One. 2011 Dec 28;6(12):e29388. doi: 10.1371/journal.pone.0029388 (PMC3247262; doi:10.1371/journal.pone.0029388)

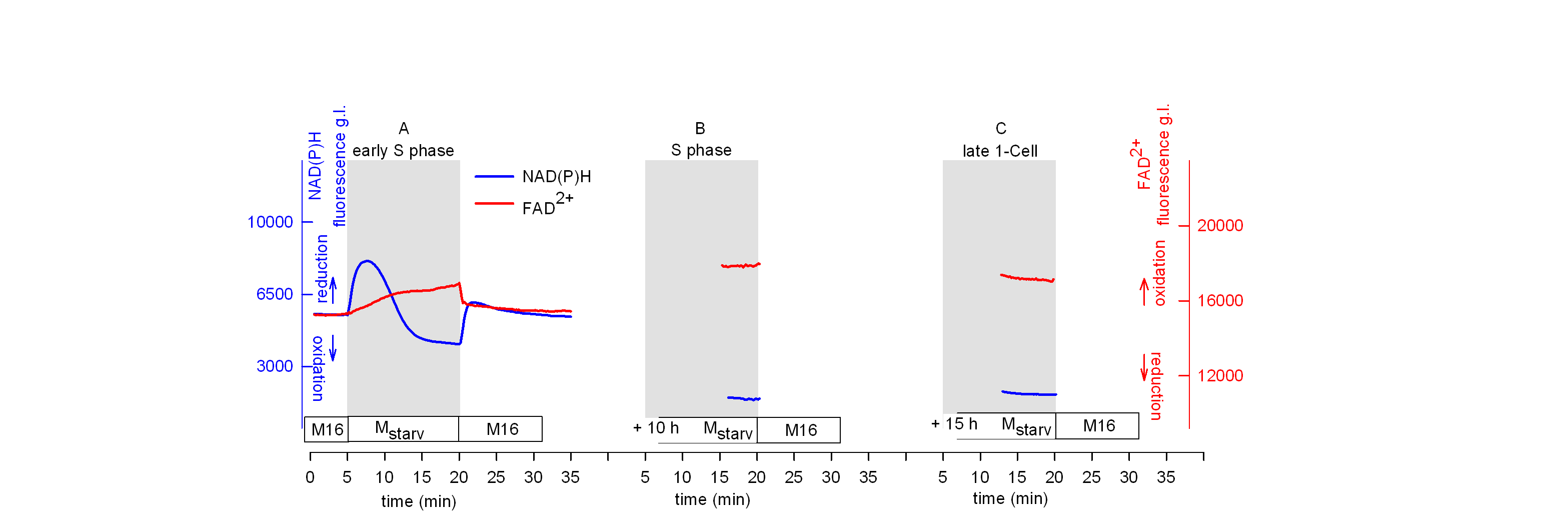

Supplement: Figure S1 — NAD(P)H and FAD2+ profiles from eggs subjected to Mstarv for 10 or 15 h. The NAD(P)H oxidation (blue line) induced by starvation in early 20 min is thereafter amplified by prolonged duration for 10 or 15 h. The level of FAD2+ (red line) is oxidized in early 20 min and remains oxidized at similar levels for 10 or 15 h. The records of the early 20 min are the copy from those plotted on Fig. 1A . (TIF) [file pone.0029388.s001.tif]

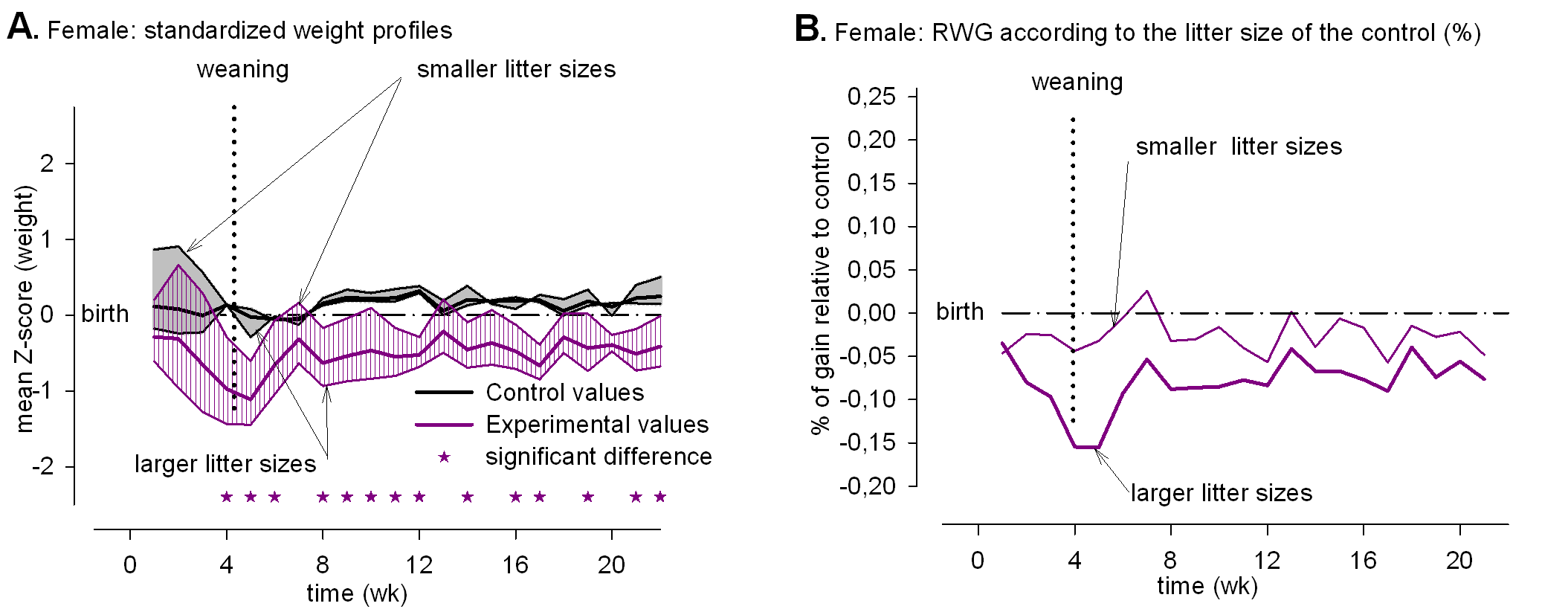

Supplement: Figure S2 — Z profiles and RWG for females issued from eggs subjected to Mstarv. (A) The upper limits of the shaded zones (grey for controls and dark pink for the experimental values) are the normalized weight profiles of females issued from the larger litter sizes (8, 7 and 6) and the lower limits, from the smaller litter sizes (5, 4, 3 and 2). (B) Relative weight gains (RWG) of experimental animals according to the larger or smaller litter size group. In this figure and in figures S4 and S8 , the stars in panel A denote significant differences between average experimental and control values (with a P-value at least <0.04) when compared to controls. (TIF) [file pone.0029388.s002.tif]

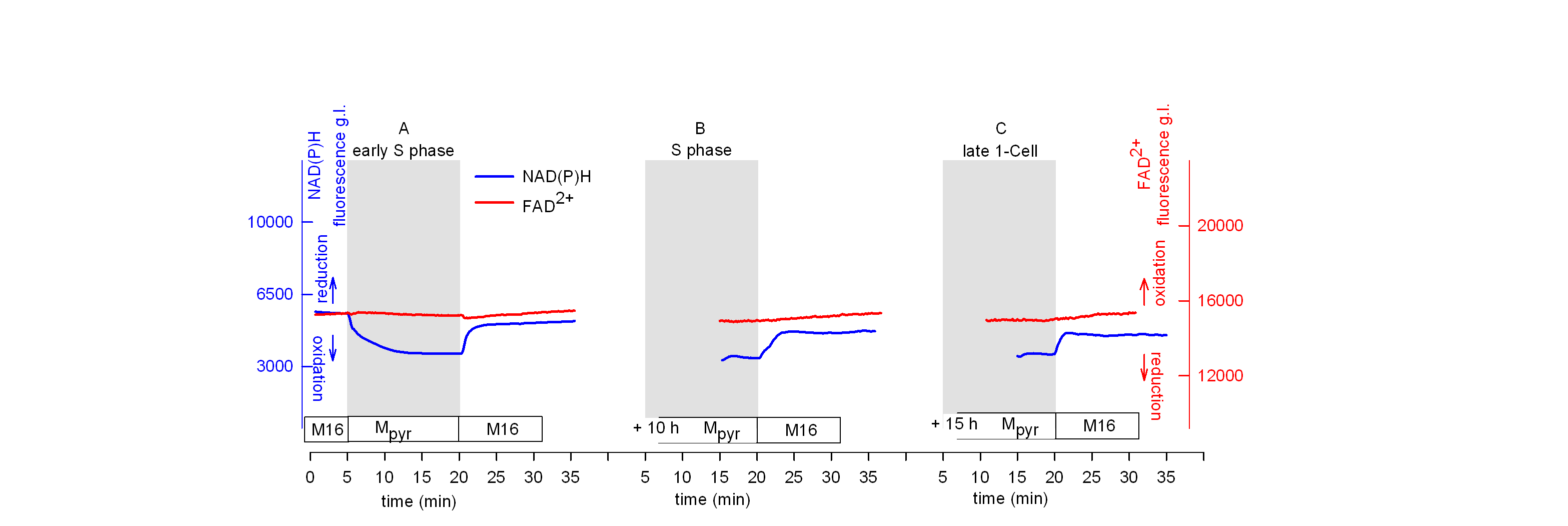

Supplement: Figure S3 — NAD(P)H and FAD2+ profiles from eggs subjected to Mpyr for 10 or 15 h. The level of NAD(P)H oxidation (blue line), induced by Mpyr in early 20 min, remains highly oxidized for 10 or 15 h. The level of FAD2+ (red line) remains at a constant level for 10 or 15 h. The records of the early 20 min are the copy from those plotted on Fig. 2A . (TIF) [file pone.0029388.s003.tif]

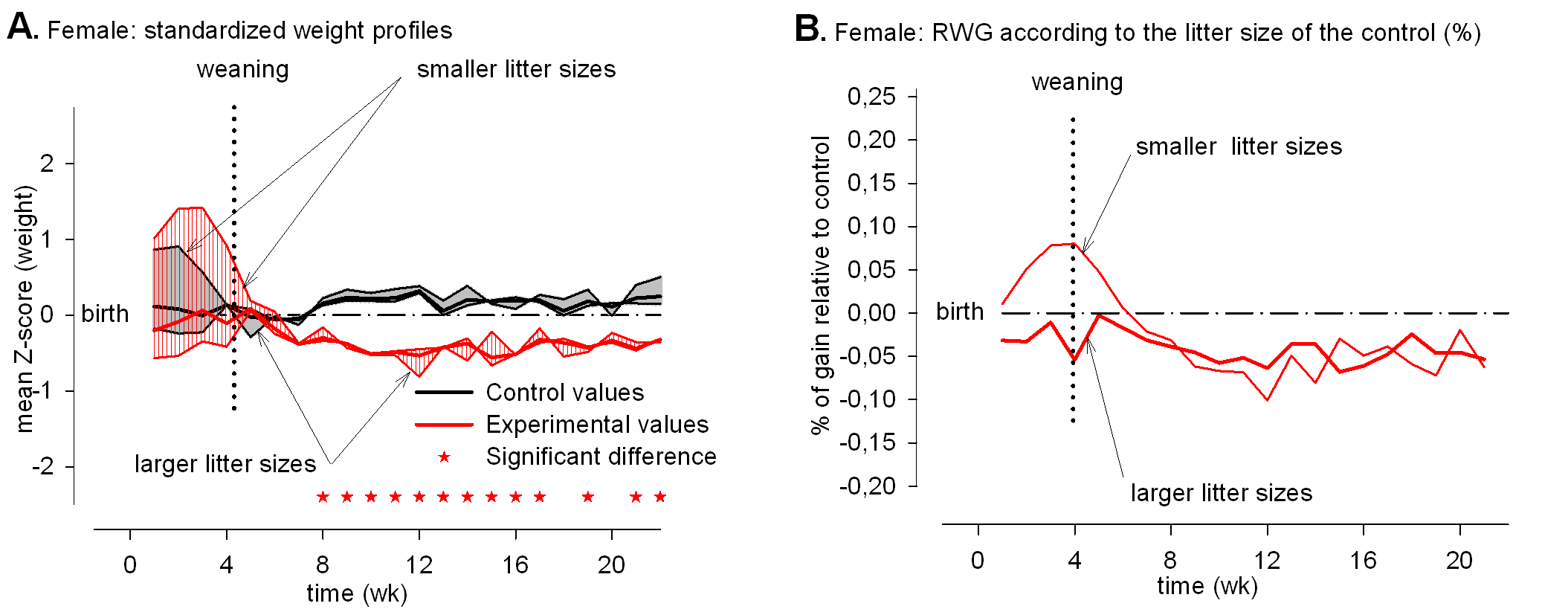

Supplement: Figure S4 — Z profiles and RWG for females issued from eggs subjected to Mpyr. (A) The upper limits of the shaded zones (grey for the controls and red for the experimental values) are the normalized weight profiles of females issued from the larger litter sizes (8, 7 and 6) and the lower limits, from the smaller litter sizes (5, 4, 3 and 2). (B) Relative weight gains (RWG) of experimental animals according to the larger or smaller litter size group. (TIF) [file pone.0029388.s004.tif]

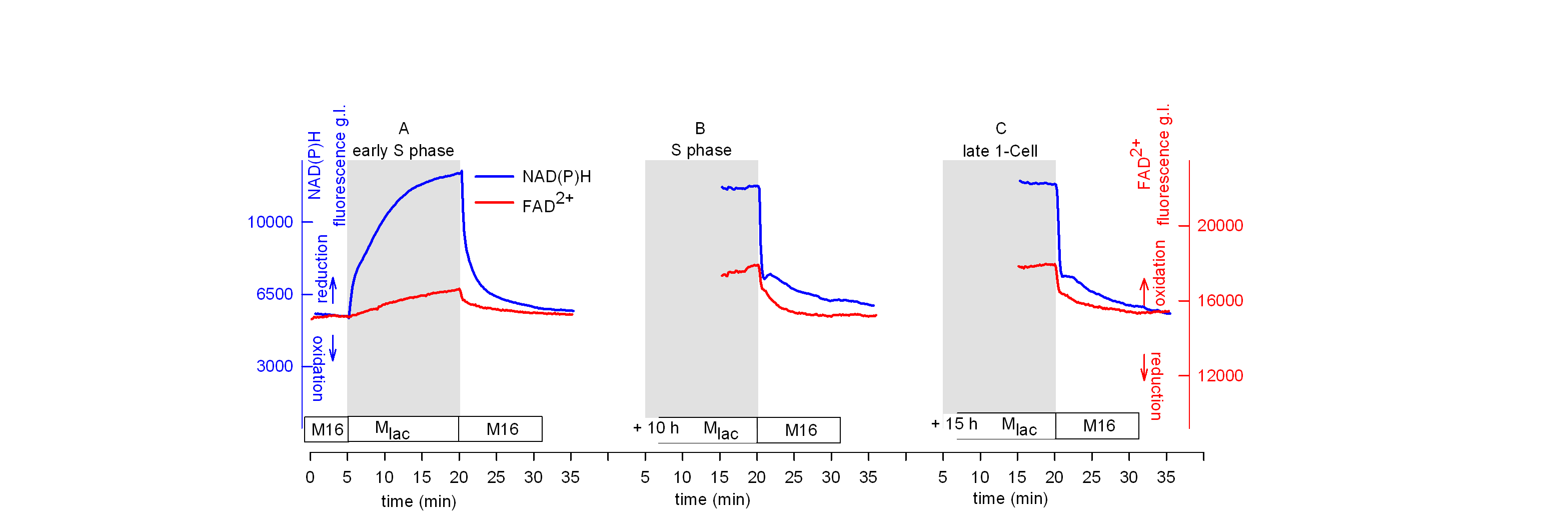

Supplement: Figure S5 — NAD(P)H and FAD2+ profiles from eggs subjected to Mlac for 10 and 15 h. The level of NAD(P)H reduction (blue line), induced by Mlac in early 20 min, remains highly reduced for 10 or 15 h. The level of FAD2+ (red line) is oxidized in the early 20 min and remains oxidized at a constant level for 10 or 15 h. The records of the early 20 min are the copy from those plotted on Fig. 3A . (TIF) [file pone.0029388.s005.tif]

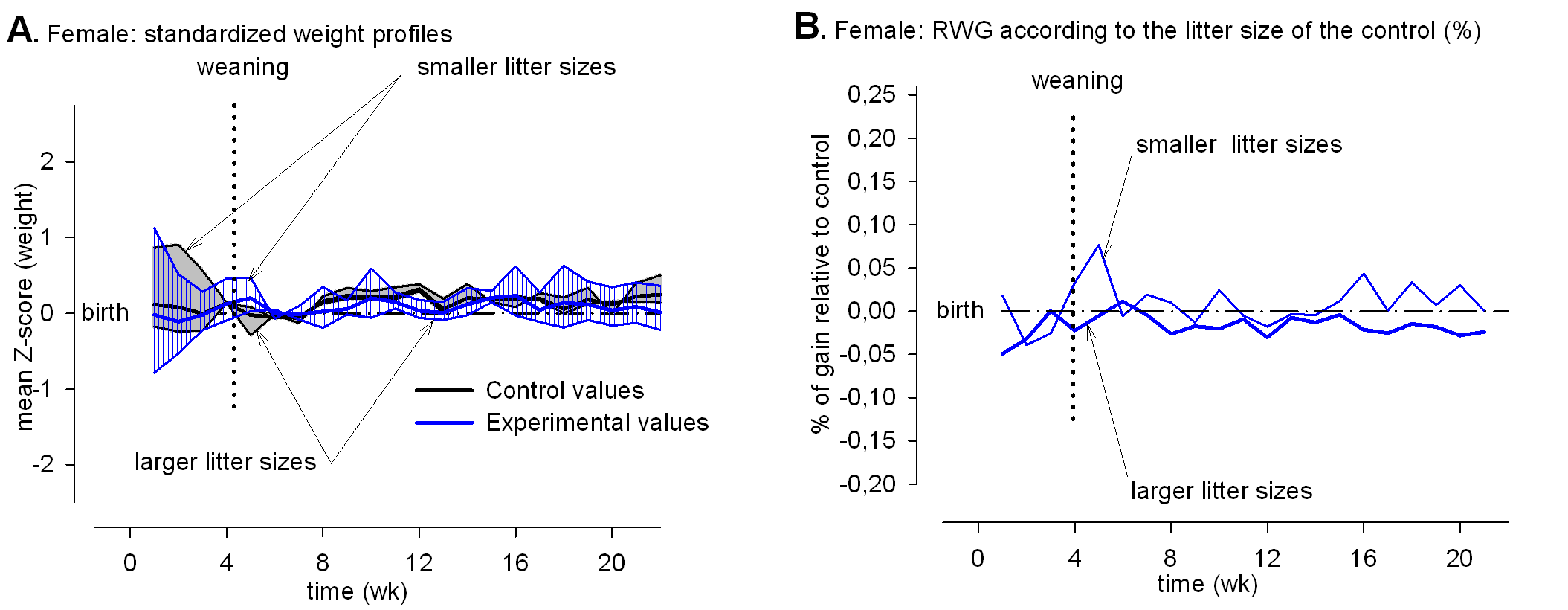

Supplement: Figure S6 — Z profiles and RWG for females issued from eggs subjected to Mlac. (A) The upper limits of the shaded zones (grey for the controls and shaded blue for the experimental values) are the normalized weight profiles of females issued from the larger litter sizes (8, 7 and 6) and the lower limits, from the smaller litter sizes (5, 4, 3 and 2). (B) Relative weight gains (RWG) of experimental animals according to the larger or smaller litter size group. (TIF) [file pone.0029388.s006.tif]

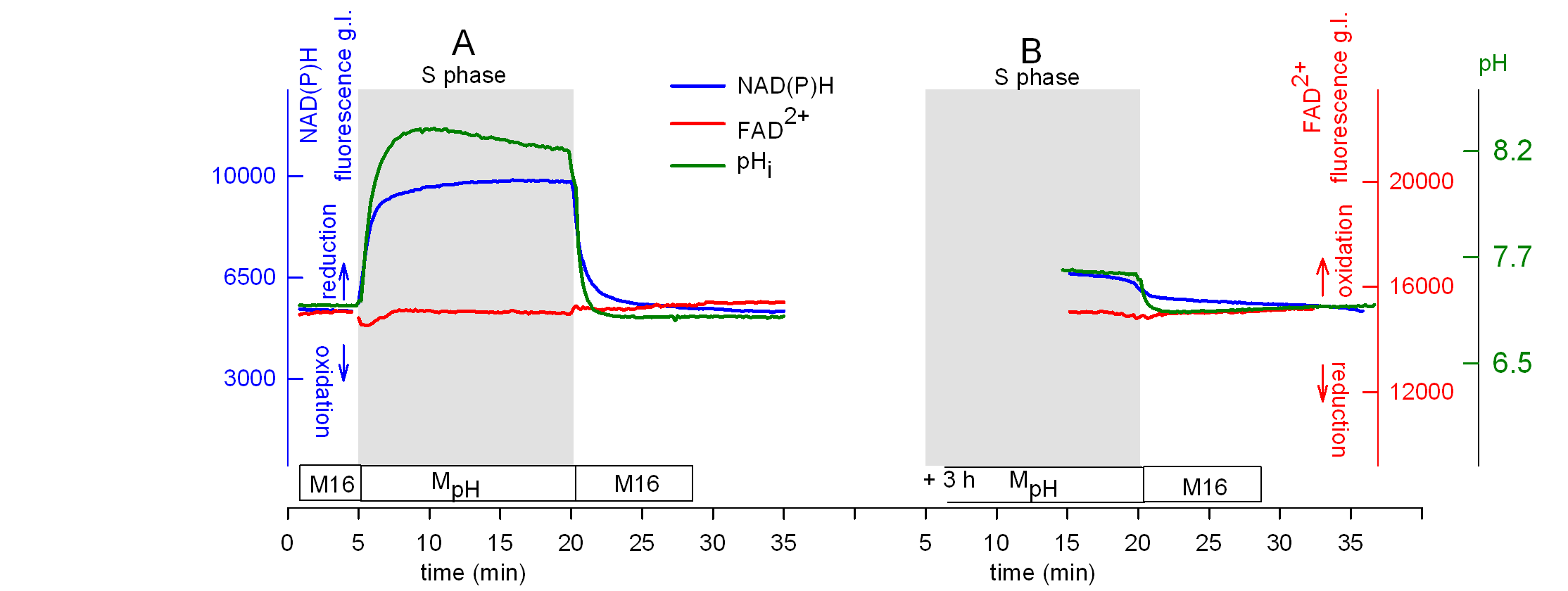

Supplement: Figure S7 — NAD(P)H and FAD2+ profiles from eggs subjected to MpH for 3 h. The records show that the NAD(P)H level induced by MpH by the early 20 min, declines rapidly thereafter and reaches the resting level in a couple of hours. The level of FAD2+ remains constant. (TIF) [file pone.0029388.s007.tif]

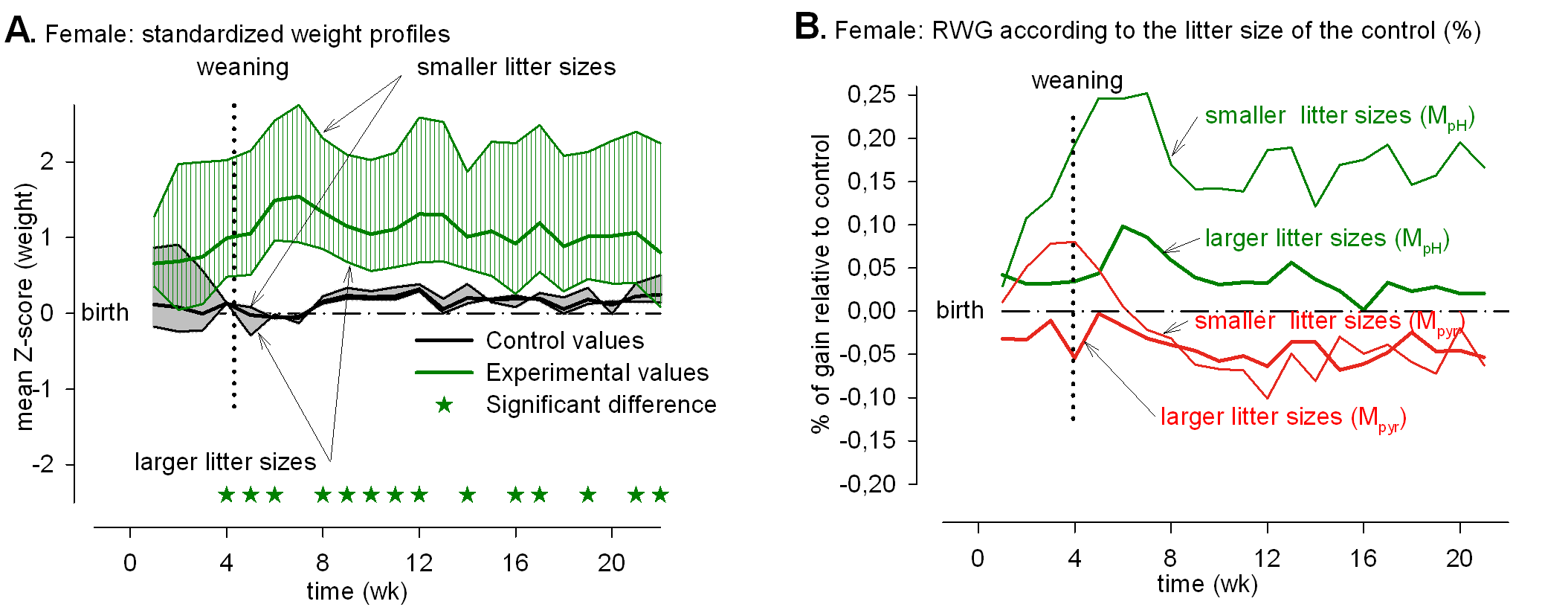

Supplement: Figure S8 — Z profiles and RWG for females issued from eggs subjected to MpH. (A) Standardized plots of total Z values (n = 210). The upper limits of the shaded zones (grey for the controls and green for the experimental values) are the normalized weight profiles of females issued from the larger litter sizes (8, 7 and 6) and the lower limits, from the smaller litter sizes (5, 4, 3 and 2). (B) Relative weight gains (RWG) of MpH experimental animals according to the larger or smaller litter size group (green lines). The RWG of Mpyr females from Fig. S4 are plotted as red lines for comparison. (TIF) [file pone.0029388.s008.tif]
